# Anemia and iron deficiency in patients with atrial fibrillation

**DOI:** 10.1186/s12872-022-02633-6

**Published:** 2022-05-04

**Authors:** Nicole Hanna-Rivero, Samuel J. Tu, Adrian D. Elliott, Bradley M. Pitman, Celine Gallagher, Dennis H. Lau, Prashanthan Sanders, Christopher X. Wong

**Affiliations:** 1 Centre for Heart Rhythm Disorders, University of Adelaide, Adelaide, Australia; 2 Department of Cardiology, Royal Adelaide Hospital, Port Road, Adelaide, 5000 Australia

**Keywords:** Atrial fibrillation, Anemia, Iron-deficiency

## Abstract

Atrial fibrillation (AF) is the most common cardiac tachyarrhythmia and has a rising global prevalence. Given the increasing burden of AF-related symptoms and complications, new approaches to management are required. Anemia and iron deficiency are common conditions in patients with AF. Furthermore, emerging evidence suggests that the presence of anemia may be associated with worse outcome in these patients. The role of anemia and iron deficiency has been extensively explored in other cardiovascular states, such as heart failure and ischemic heart disease. In particular, the role of iron repletion amongst patients with heart failure is now an established treatment modality. However, despite the strong bidirectional inter-relationship between AF and heart failure, the implications of anemia and iron-deficiency in AF have been scarcely studied. This area is of mechanistic and clinical relevance given the potential that treatment of these conditions may improve symptoms and prognosis in the increasing number of individuals with AF. In this review, we summarise the current published literature on anemia and iron deficiency in patients with AF. We discuss AF complications such as stroke, bleeding, and heart failure, in addition to AF-related symptoms such as exercise intolerance, and the potential impact of anemia and iron deficiency on these. Finally, we summarize current research gaps on anemia, iron deficiency, and AF, and underscore potential research directions.

## Background

Atrial fibrillation (AF) is the most prevalent cardiac tachyarrhythmia encountered in clinical practice [1, 2]. In 2010, it was estimated that there were already 33 million people with AF worldwide, and projections suggest this may exceed 70 million in Asia alone by 2050 [2, 3]. Complications of AF include an elevated risk of stroke, heart failure, dementia, and premature death [4]. Additionally, associated symptoms include chest pain, palpitations, fatigue, dizziness, syncope, dyspnea, and a decrease in exercise capacity [5]. Furthermore, recent data suggest that there is an increasing burden of AF-related symptoms and complications on healthcare systems. For example, hospitalizations for AF in Australia have demonstrated a 295% increase in recent years and now represent the most common cause for cardiovascular hospitalisation [6, 7]. These trends are concerning and suggest that there is a need to evaluate current management approaches and investigate new treatments.

Anemia is common in patients with AF [8]. As with AF, the prevalence of anemia increases with rising age [9]. An increasing number of studies have demonstrated that the presence of anemia is associated with increased risks of bleeding, cardiac events, and overall mortality in patients with AF [8–11]. However, the reasons for these associations remain unclear and have not been adequately explored. Recent cohort studies have also shown that lower hemoglobin levels and anemia may be associated with the development of new-onset AF [12, 13]. Furthermore, patients with anemia may also be more likely to experience clinical recurrence of AF [14]. Anemia is also prevalent in other cardiac conditions where it has been more comprehensively characterised, and is frequently caused by iron deficiency. For example, in chronic heart failure, the prevalence of iron deficiency has been shown to be approximately 20–30% [15, 16]. Additionally, heart failure patients with iron deficiency have deleterious symptoms and cardiac outcomes, independent of anemia status [17]. However, despite the increasing prevalence of both AF and anemia with ageing populations, information on the prevalence and impact of anemia and iron deficiency in patients with AF remains limited.

In this review, we thus sought to summarise the current published literature on anemia and iron deficiency in patients with AF. We discuss AF complications such as stroke, bleeding, and heart failure, in addition to associated symptoms such as exercise intolerance, and the potential impact of anemia and iron deficiency on these. Finally, we summarize current research gaps on anemia, iron deficiency, and AF, and underscore potential research directions. Currently, anemia and iron deficiency are not routinely screened for or treated in patients with AF, compared to a more established role in heart failure management. Current international guidelines for heart failure management advise routine screening and treatment for iron deficiency and anemia to improve functional capacity, reduce hospitalisations, and treat related symptoms [18]. This review suggests further study in this area of research may potentially clarify whether routine screening and treatment for anemia and iron deficiency in patients with AF may improve symptoms and reduce the risk of complications.

## Outcomes associated with AF

The presence of AF can lead to numerous complications with significant clinical impact. An increased risk of stroke, heart failure, renal disease, dementia, and premature mortality are amongst the most notable clinical concerns associated with AF [19]. Concerningly, the occurrence of stroke can be the first presenting symptom of AF in 15–20% of patients and contribute to future disability and cognitive decline [5]. Reassuringly, the risk of stroke can be reduced by approximately 70% with appropriate anticoagulation therapy [20]. Although outweighed by the benefits of stroke risk reduction, potential side effects of anticoagulants include an increased risk of both intracranial and extracranial bleeding [21]. Gastrointestinal bleeding may be one likely mechanism by which patients with AF are more susceptible to the development of iron deficiency and anemia.

## Symptoms associated with AF

Although there is often a focus on associated complications, patients with AF can experience a diverse range of symptoms that significantly impacts on quality of life [5]. Typical symptoms associated with AF include chest pain, palpitations, dyspnea, fatigue, exercise-intolerance, dizziness, and syncope. A majority of individuals will experience one or more of these symptoms attributable to AF at some point in time [5]. However, as many as 25–30% of patients may ostensibly not experience, or more likely not recognize, AF-related symptoms that may limit earlier diagnosis and treatment [22]. There is also a high proportion of co-existing cardiac diseases amongst patients with AF, such as heart failure and valvular disease. These conditions may also present with similar symptoms and can thus delay the diagnosis of paroxysmal AF or confound the assessment of AF-related symptoms [5]. There is also established evidence that exercise intolerance is highly prevalent in patients with AF [5]. More than half of all AF patients experience a reduced exercise capacity in the order of 15–20%, and this can lead to symptoms of dyspnea, fatigue, and a poorer quality of life [5].

Despite being a major contributor to impaired quality of life and healthcare presentation, the mechanisms underlying AF symptoms are largely under-researched. Although many individuals experience palpitations, the pathways leading to this sensation remain unclear [23]. Chest pain often occurs in patients with AF, and this is generally attributed to a reduction in coronary perfusion due to rapid and irregular ventricular rates [24]. Exercise limitation, dyspnea and fatigue may be caused by multiple mechanisms. AF can disrupt normal hemodynamics by impairing diastolic filling, causing a loss of atrioventricular synchrony, and predisposing to cardiomyopathy [25–28]. In non-AF patients, anemia is associated with increased cardiac output and workload and left ventricular hypertrophy [29]. Both anemia and AF are independently associated with alterations in cardiac function and output, yet interactions with the co-existence of both conditions have yet to be studied. Although exercise performance depends on both cardiac output and oxygen transport, there has been little investigation into the impact and mechanisms of anemia and iron deficiency on AF-related symptoms, discussed further below.

## Symptomatic treatment for AF

Physical tests for exercise capacity have been utilized as a measure of assessing improvement in AF symptoms, cardiac events, and overall quality of life. For example, it has been shown that increases in exercise performance associated with maintenance of sinus rhythm correlate with physical activity, functional capacity, and overall vitality [30]. Furthermore, exercise capacity was shown to be a significant predictor of emotional as well as physical health [30].

Studies for exercise training have shown a substantial benefit in the management of heart failure. The HF-ACTION clinical trial, which involved 1620 heart failure patients, demonstrated that every incremental increase of 6% in peak oxygen consumption (VO2) can result in a 5% improvement in all-cause mortality and rates of hospitalization [31]. Based on the above, investigators have subsequently evaluated the effect of introducing supplementary exercise programs on AF-related symptoms and quality of life. One study assessed a 3-month exercise training program in AF patients and controls. Despite a lower peak oxygen uptake in the AF group, training resulted in a similar increase in oxygen uptake in both groups [32]. A reduction in resting heart rate was also observed in the AF cohort, suggesting that improving exercise performance may improve AF rate control [32]. Another study using an 8-week training program showed a positive correlation between exercise performance and outcomes of physical functioning, bodily pain, vitality, and emotion at 8-weeks post baseline [33].

Finally, it has also been shown that rhythm control for AF can subsequently improve exercise capacity. For example, two studies that studied patients with persistent AF treated with catheter ablation reported a significant improvement in exercise performance when sinus rhythm was restored [34, 35]. Pharmacological strategies using anti-arrhythmic medication for restoration of sinus rhythm can additionally improve exercise capacity and overall vitality in persistent AF patients [36].

In contrast, despite the evidence suggesting their relevance outlined below, there is no data on the role of anemia and iron deficiency management for symptom improvement in patients with AF.

## Anemia and iron deficiency

Anemia is a hematological condition that results from a deficiency of red blood cells and is most commonly defined as a reduced hemoglobin concentration [37]. Estimates of anemia prevalence vary between sex and age. The worldwide prevalence of anemia is estimated to be 30% in non-pregnant women, 12% of all men, and 24% of the elderly [38]. Anemia prevalence also increases exponentially with increasing age. In Australia and the United States, the prevalence of anemia is approximately 10% in young non-pregnant women, 11% in males aged ≥ 65 years, and 20% in those aged 85 years or older [39–41]. Amongst various causes, anemia due to iron deficiency is most common. Similarly, the prevalence of iron deficiency varies by ethnicity, sex, and age. In developing countries, the prevalence of iron deficiency can be as high as 41–63% in women and 13% in men, whilst iron deficiency anemia may be present in 20–39% of women and 4% of men [42]. However, as comprehensive data is limited, it is possible that the true burden of iron deficiency, particularly non-anemic iron deficiency, may be underestimated by these figures.

In addition, there are numerous definitions and guidelines for diagnosing iron deficiency [43]. An indication of iron stores can be obtained from serum iron, ferritin, and transferrin levels [44]. However, elevated levels of serum ferritin also occur in other chronic inflammatory conditions independently of iron status due to the nature of ferritin as an acute phase reactant. Production of serum ferritin increases 2.5-fold in chronic inflammatory states [45, 46]. To better evaluate for iron-deficiency in these cases, additional evidence of a low transferrin saturation under 20% and an assessment of inflammatory markers (such as erythrocyte sedimentation rate and C-reactive protein) are indicated [47, 48]. Notably, a ferritin level of less than 100 ug/L, or a combination of a ferritin level between 100 and 299 ug/L and transferrin saturation under 20%, are now guideline-recommended cut-offs for diagnosing iron deficiency in patients with heart failure, as discussed below [49].

## Anemia and iron deficiency in systolic heart failure

Iron supplementation is a now an established treatment for heart failure patients with comorbid iron deficiency. Up to half of all heart failure with reduced ejection fraction patients have evidence of iron deficiency [50], and the prevalence may be even higher in patients with heart failure with preserved ejection fraction, reaching almost 60% [51]. Furthermore, this high prevalence of iron deficiency occurs even in the absence of anemia [16, 17, 48, 52]. Although treatment of anemia and iron deficiency with subcutaneous erythropoietin and oral iron supplementation has not been shown to be beneficial, trials of intravenous iron repletion have demonstrated consistent benefits [50]. The FAIR-HF trial that recruited heart failure patients with iron-deficiency showed a significant benefit of intravenous ferric carboxymaltose compared to placebo for improvements in patient global assessment, 6-min walk test, and quality of life [50]. Importantly, these benefits were similar regardless of whether patients were anemic. The CONFIRM-HF trial also demonstrated that ferric carboxymaltose compared to placebo resulted in improvements in 6-min walk test, New York Heart Association class, patient global assessment, and health-related quality of life [50]. Finally, the EFFECT-HF trial studied the effect of intravenous iron supplementation on peak oxygen consumption and found that the treatment group were able to maintain peak oxygen consumption, compared to a reduction in the control group, at 24 weeks follow up [53]. A meta-analysis pooling data trials on intravenous iron therapy compared to placebo concluded that intravenous iron in chronic heart failure reduced cardiac related hospitalizations, cardiac-related mortality, and all-cause mortality [54]. More recently, the AFFIRM-AHF trial demonstrated that intravenous iron supplementation also reduced recurrent hospitalizations after recent discharge following acute heart failure [55].

## Mechanisms of iron deficiency in other chronic inflammatory diseases

**Fig. 1:**
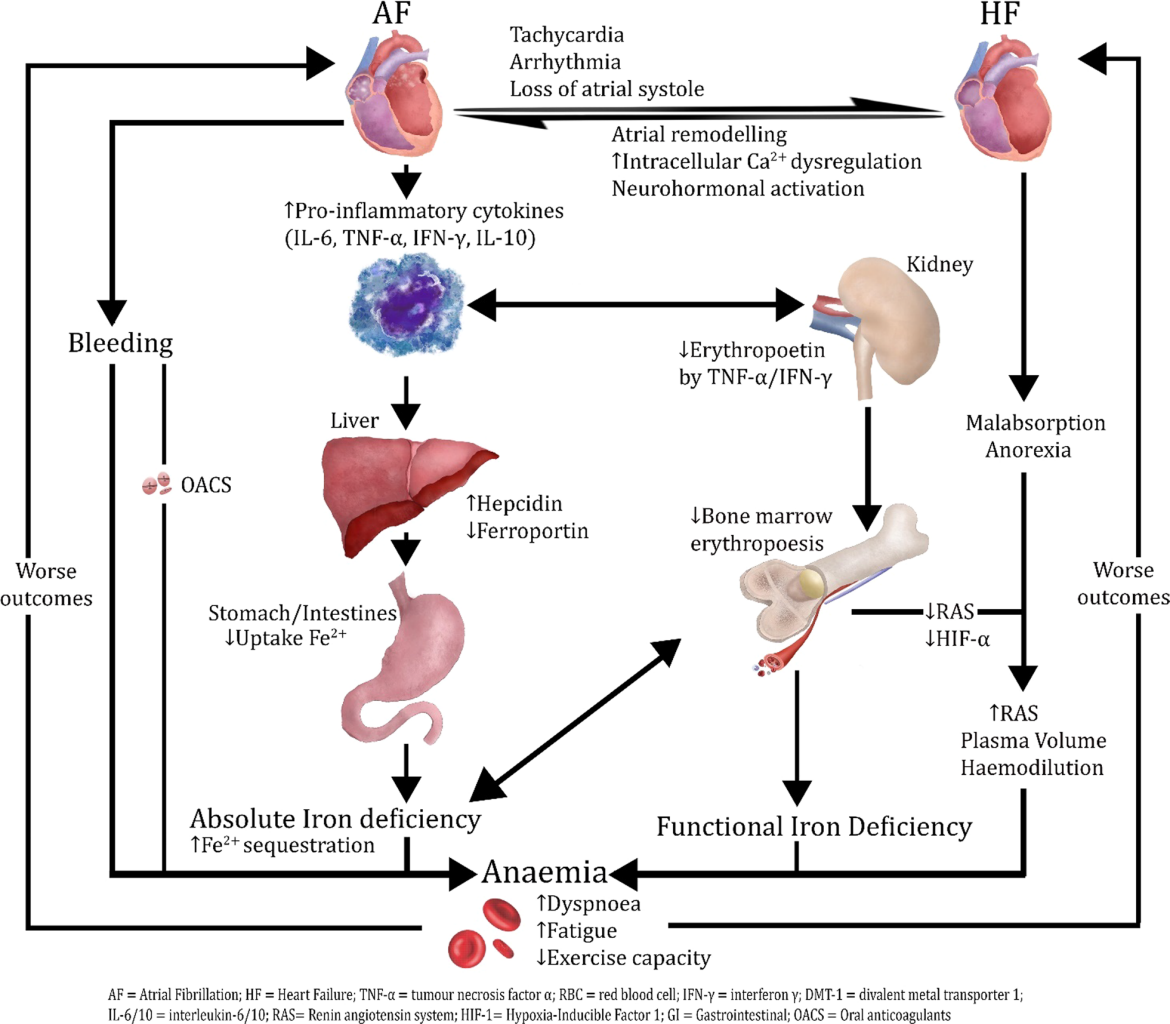
Potential mechanisms linking atrial fibrillation, heart failure, iron deficiency, and anemia

Iron supplementation is thought to be beneficial in-part because heart failure, similar to chronic kidney disease, cancer, and other inflammatory disorders, is associated with an increase in systemic inflammation and subsequent abnormal homeostasis of systemic iron (Fig. 1) [56, 57].

Our understanding of potential mechanisms for this phenomenon, termed functional iron deficiency, has been advanced by several observations in chronic inflammatory conditions. Proinflammatory cytokines such as interleukin-6 mediate the production and release of hepcidin [58]. Hepcidin is a peptide that regulates iron storage and release by mediating activity of ferroprotein, an iron transporter protein [50]. In chronic inflammatory states, there is an over-expression of hepcidin which predominantly contributes to iron deficiency by decreasing ferroprotein and results in a trapping of iron in duodenal enterocytes and macrophages [50, 56]. Furthermore, interferon-γ, lipopolysaccharide and tumour necrosis factor-α upregulate the expression of divalent metal transporter 1, which increases the uptake of iron by macrophages [56]. Thus, an abnormally greater uptake and retention of iron occurs within the storage cells of the reticuloendothelial system, which limits iron availability for erythroid progenitor cells and erythropoiesis. Finally, inflammatory cytokines such as interleukin-1 and tumour necrosis factor-α inhibit erythropoietin expression and increase erythrophagocytosis, thus also contributing to eventual anemia [59].

Thus, chronic inflammatory conditions such as heart failure result in a multi-faceted pathophysiological process that precedes the development of anemia by reducing the amount available iron for erythropoiesis. This reduced availability of iron underpins the benefit of intravenous iron supplementation in heart failure patients [60]. Notably, oral supplementation of iron has not been shown to be beneficial in contrast to intravenous replacement. Oral dosing of iron typically has poor compliance, has limited gastrointestinal absorption through mucosal edema, and is thus insufficient to counteract the sequestration of stored iron out of enterocytes and macrophages [61].

## Potential role for anemia and iron deficiency in atrial fibrillation

The role of anemia and iron-deficiency is well-established in patients with heart failure. However, there has been comparatively less research into the potential impact of these conditions in patients with AF. This is despite the fact that AF and heart failure commonly co-exist, share key risk factors, have similar pathophysiological mechanisms, and predispose to each other (Fig. 1). Both conditions experience similar consequences of a reduction in cardiac output, oxygen intake and peak work load [62]. Furthermore, emerging evidence suggests a significant role of inflammation in the pathogenesis of AF. For example, studies have shown heightened activity from lymphomononuclear cells, death of myocytes, elevated inflammatory markers and elevated neutrophil/lymphocyte ratios in patients with lone AF compared to control patients without AF [63]. Signalling pathways existent in inflammatory states can also inflict atrial remodeling and restrict atrial conduction through mediation of matrix metalloproteinases (MMP) 2 and MMP-9 [63]. Finally, anemia and iron-deficiency themselves can lead to significant myocardial hypertrophy and chamber dilatation, which can predispose to both heart failure and AF [64]. Thus, the interrelationship of AF, heart failure, and inflammation raise the strong possibility that associations of anemia and iron-deficiency in AF may be similar to that seen in heart failure.

## Existing data on anemia and atrial fibrillation

To date, there are a limited number of studies evaluating the prevalence of anemia in AF patients (Table 1). A sub-study using the AFCAS registry found 30% of AF patients with available hemoglobin determinations undergoing coronary artery stenting to be anemic [65]. This prevalence rate was similar to another Danish registry study finding a prevalence rate of 34% in a cohort of over 18,000 AF patients [9]. In contrast, the prevalence was found to be 12% in another sample, suggesting there is some variation amongst different AF populations [11]. In a recent systematic review and meta-analysis of available data from 28 studies, we found the weighted proportion of AF patients with anemia to be 16% [8].

**Table 1:** Studies reporting prevalence and associations of anemia or iron deficiency amongst individuals with AF

Study	Study type	Study size	Population type	Anemia, n (%)	Iron deficiency, n (%)	Outcomes studied
Shireman, 2006 [77]	Cohort study	26,345	Inpatient	2107 (8)	–	Bleeding
Sharma, 2009 [78]	Cohort study	13,067	Inpatient	7056 (54)	–	All-cause mortality
Park, 2011 [79]	Cohort study	488	Outpatient	–	–	All-cause mortality
Suzuki, 2012 [80]	Cohort study	1942	Inpatient	94 (5)	–	Mortality and hospitalization
Lip, 2012 [81]	Cohort study	7156	Inpatient	71 (1)	–	Bleeding
Friberg, 2012 [82]	Cohort study	90,490	Inpatients and outpatients	–	–	Bleeding
Manzano-Fernandez, 2012 [83]	Cohort study	285	Inpatient	105 (37)	–	Bleeding
Takabayashi, 2014 [84]	Cohort study	2774	Outpatient	471 (17)	–	Hospitalization and major bleeding
Puurunen, 2014 [65]	Cohort study	861	Inpatient	258 (30)	–	Bleeding and composite all-cause mortality, non-fatal MI, TIA, stroke, bleeding
Goodman, 2014 [85]	Secondary analysis of RCT	14,236	Anticoagulant trial	1976 (14)	–	Bleeding
Westenbrink, 2015 [10]	Secondary analysis of RCT	17,796	Anticoagulant trial	2223 (13)	–	Thromboembolism, all-cause mortality, MI, bleeding, stroke
Sherwood, 2015 [86]	Secondary analysis of RCT	14,236	Anticoagulant trial	1993 (14)	–	Bleeding
Lee, 2015 [87]	Cohort study	166	Outpatient	54 (33)	–	Mortality and hospitalization for heart failure
O’Brien, 2015 [88]	Cohort study	7411	Outpatient	2742 (37)	–	Bleeding
Dodson, 2016 [89]	Cross sectional study	31,951	Inpatients and outpatients	6514 (20)	–	Bleeding
Hori, 2016 [90]	Secondary analysis of RCT	1278	Anticoagulant trial	–	–	Bleeding
Kobayashi, 2016 [91]	Cohort	227	Inpatient	104 (46)	–	Bleeding
Li, 2016 [92]	Cohort	4632	Inpatients and outpatients	324 (7)	–	All-cause mortality
Westenbrink, 2017 [11]	Secondary analysis of RCT	18,103	Anticoagulant trial	2288 (12.5)	–	All-cause mortality, major bleeding, stroke, systemic embolism
Aisenberg, 2018 [93]	Secondary analysis of RCT	21,026	Anticoagulant trial	9885 (47)	–	Bleeding
Perera, 2018 [94]	Secondary analysis of RCT	7554	Anticoagulant trial	1888 (25)	–	All-cause mortality
Keskin, 2018 [73]	Cohort study	101	Outpatient	31 (30.7)	48 (47.5)	ID/Haematinic deficiencies compared to control group
Bonde, 2019 [9]	Cohort study	18,734	Inpatient	6358 (34)	–	Stroke, thromboembolism, major bleeding
An, 2019 [95]	Cohort study	4169	Outpatient	1547 (37)	–	Stroke, systemic embolism, bleeding, and mortality
Tiili, 2019 [96]	Cohort study	53,953	Outpatient	1619 (3)	–	Bleeding
Kuronuma, 2019 [97]	Cohort study	3237	Outpatient	–	–	All cause, cardiovascular and non-cardiovascular mortality
Fu, 2019 [98]	Cohort study	219	Inpatient	–	–	
Kodani, 2020 [99]	Cohort study	6536	Outpatient	1015 (16)	–	Thromboembolism, all-cause mortality
An, 2020 [69]	Cohort study	4169	Outpatient	1547 (37)	–	Thromboembolism, bleeding, heart failure
Krittayaphong, 2021 [67]	Cohort study	1562	Outpatient	518 (33)	–	Thromboembolism, bleeding, heart failure, mortality
Minhas, 2021 [68, 76, 100]	Cross-sectional	5,975,241	Inpatient	–	152,059 (3)	Length of stay, hospitalization costs, myocardial infarction, vasopressor use, mechanical ventilation, kidney injury, mortality
Al-Hussainy, 2021 [101]	Cohort study	41,321	Outpatient	11,812 (29)	–	Stroke, bleeding
Hashimoto, 2021 [68]	Cohort study	1677	Outpatient	378 (23)	–	Heart failure, bleeding, quality of life
Zhang, 2022 [100]	Cohort study	18,106	Outpatient	2500 (14)	–	Bleeding, cardiovascular and all-cause mortality

MI, myocardial infarction; TIA, transient ischemic attack, ID, iron-deficient, RCT, Randomised Control Trial

The AFCAS registry study found anemia to be an independent predictor for all-cause mortality, major adverse cardiac and cerebrovascular events [65]. The Danish registry study also showed anemia to be significantly associated with increased risks of major bleeding events, stroke, thromboembolic events and all-cause mortality compared to non-anemic AF patients [9]. Two analyses on individuals in different oral anticoagulant control trials also showed associations with anemia and increased risks in major bleeding and all-cause mortality [10, 11, 66]. In our recent meta-analysis, we found anemia to be associated with a 78% increased hazard of all-cause mortality, and every 1 g/dL decrease in hemoglobin associated with a 24% increased hazard [8]. Furthermore, anemia was associated with a 15% increased hazard of stroke or systemic thromboembolism, and 78% increased hazard of major bleeding. More recently, several cohort studies have also reported associations of anemia with greater risks of heart failure hospitalization [67–69]. Furthermore, at least one study has also suggested that anemia may associated with clinical recurrence of AF [14]. In this large cohort, patients with anemia had greater post-ablation AF compared to patients without anemia. Although this association requires replication, it seems plausible that this could be due to an adverse effect of anemia on atrial remodeling. Despite these associations persisting after multivariate adjustment and being robust to a range of sensitivity analyses, it should be acknowledged that these studies are observational in nature, and as such, may be subject to residual or unmeasured confounding. Nonetheless, these studies on anemia in AF patients raise the potential relevance of anemia to subsequent complications. However, there are limited studies assessing anemia on other AF symptoms, such as functional capacity and exercise tolerance. One recent study reported quality of life using a validated questionnaire amongst AF patients with and without anemia. While baseline quality of life did not appear to differ, those with anemia did not demonstrate as much quality of life improvement at follow-up compared to non-anemic patients [68].

The aforementioned associations have biologic plausibility. For example, an effect of anemia on mortality could feasibly be mediated by established associations of anemia with other deleterious outcomes such as stroke, heart failure, and bleeding [70–72]. Conversely anemia may also be a marker of general frailty and the latter a potential confounder. However, given the magnitude of reported associations, we would argue that further investigation into the prognostic implications of anemia in AF, and whether treatment of this comorbidity may be beneficial, is warranted.

## Existing data on iron deficiency and atrial fibrillation

There is a paucity of data on iron deficiency in patients with AF (Table 1). One retrospective study in Turkey has been published that analysed data on iron deficiency in 101 patients with AF [73]. Using a diagnostic threshold of ferritin level less than 100 ug/L, or a combination of a ferritin level between 100 and 299 ug/L and transferrin saturation under 20%, these investigators found that 47.6% of individuals with AF had iron deficiency [73]. Furthermore, the prevalence appeared to be greater in patients with permanent AF compared to those with paroxysmal or persistent AF. Limitations include the small nature of the study and absence of medication data such as anticoagulant use [74, 75]. More recently, a large analysis of the National Inpatient Sample was undertaken whereby 2.5% of hospitalized AF patients had a coded diagnosis of iron deficiency anemia [76]. In cross-sectional analyses, iron deficiency anemia was associated with longer length of stay and worse inpatient outcomes (such as myocardial infarction, kidney injury, and vasopressor/ventilation requirement) but not mortality. Despite the significant size, the reported prevalence in this study is likely to be an underestimate due to the use of hospital coding data. Further studies in a variety of populations are warranted to better characterise the prevalence of iron deficiency and iron-deficiency, as does an assessment of the potential relevance of these conditions to AF symptoms and complications, as is the case in heart failure and other cardiovascular diseases.

## Clinical implications and future directions

The available evidence to date suggests that there may be a significant prevalence of anemia and iron-deficiency in individuals with AF. Furthermore, anemia appears to be associated with worse clinical outcomes in patients with AF. Data on iron deficiency in AF is limited but preliminary findings raise the possibility that prevalence of iron deficiency may not be insignificant and might also be correlated with the AF severity and clinical outcomes.

Although there is clearly a paucity of reports in this area, and these limited data should be interpreted with caution at this point in time, these initial findings raise the strong possibility that anemia and iron deficiency may be a therapeutic target in patients with AF. Furthermore, this potential is underpinned by a close interrelationship between AF and heart failure, where the role and benefit of iron supplementation is now established.

Future observational studies should provide additional estimates of anemia and iron deficiency prevalence in patients with AF, clarify associations of anemia and iron deficiency with clinical outcomes, and characterize any impact of anemia and iron deficiency on AF symptoms and functional capacity (Table 2). Importantly, these studies be done in patients with and without heart failure in order to provide some insight into the potential confounding effect of this condition. Should this line of investigation prove promising, clinical trials to correct iron deficiency may be worthwhile considering. If successful, this may lead to the routine screening and treatment of anemia and iron-deficiency in patients with AF, as has been evaluated in the heart failure sphere. Given the increasing burden of AF worldwide, anemia and iron deficiency may thus be a novel therapeutic strategy which future studies should evaluate.

**Table 2:** Future research directions

Area	Goals
Incidence and prevalence	Additional studies on the incidence and prevalence of anemia and iron-deficiency in different AF subpopulations, including by age, gender, AF type, anticoagulation use, ethnicity, and setting (outpatient, inpatient)
Symptoms	Impact of anemia and iron deficiency on AF symptoms
	Impact of anemia and iron deficiency on exercise/functional capacity and peak oxygen consumption in patients with AF
Complications	Confirmation of reported associations of anemia, stroke, and bleeding in patients with AF
	Impact of iron deficiency on future complications such as stroke, bleeding, heart failure, and mortality
Pathophysiology	Assessment of iron-deficiency and other causes as contributors to anemia in patients with AF
Therapy	Evaluation of investigation and treatment of anemia and iron-deficiency in patients with AF

AF, atrial fibrillation

## Conclusion

Both anemia and iron deficiency appear to be highly prevalent amongst individuals with AF. Furthermore, anemia and iron-deficiency may be associated with worse symptoms and outcomes in patients with AF. Although these preliminary findings require confirmation, the limited data to date support the possibility that investigation and treatment of anemia and iron deficiency may have benefit in symptomatic patients with AF. Future studies are required to confirm the prevalence of anemia and iron deficiency across different populations with AF, better characterise associations with outcomes, and ultimately determine if correction of anemia and iron-deficiency is novel management strategy in patients with AF.

## Data Availability

Not applicable to this review article.

## Abbreviations

AF: Atrial fibrillation
AFCAS: Atrial Fibrillation undergoing Coronary Artery Stenting
AFFIRM-AHF: Study to Compare Ferric Carboxymaltose With Placebo in Patients With Acute Heart Failure and Iron Deficiency
CONFIRM-HF: A Study to Compare the Use of Ferric Carboxymaltose with Placebo in Patients with Chronic Heart Failure and Iron Deficiency
EFFECT-HF: Effect of Ferric Carboxymaltose on Exercise Capacity in Patients with Iron Deficiency and Chronic Heart Failure
FAIR-HF: Ferric Carboxymaltose in Patients with Heart Failure and Iron Deficiency

## References

[1] Ball J, Carrington MJ, McMurray JJV, Stewart S (2013). Atrial fibrillation: profile and burden of an evolving epidemic in the 21st century. Int J Cardiol.

[2] Wong CX, Brown A, Tse HF, Albert CM, Kalman J, Marwick TH, Lau DH, Sanders P (2017). Epidemiology of atrial fibrillation: the Australian and Asia-Pacific perspective. Heart Lung Circ.

[3] Chugh SS, Havmoeller R, Narayanan K, Singh D, Rienstra M, Benjamin EJ, Gillum RF, Kim YH, McAnulty JH, Zheng ZJ, Forouzanfar MH, Naghavi M, Mensah GA, Ezzati M, Murray CJ (2014). Worldwide epidemiology of atrial fibrillation: a Global Burden of Disease 2010 Study. Circulation.

[4] Emdin CA, Wong CX, Hsiao AJ, Altman DG, Peters SA, Woodward M, Odutayo AA (2016). Atrial fibrillation as risk factor for cardiovascular disease and death in women compared with men: systematic review and meta-analysis of cohort studies. BMJ.

[5] Rienstra M, Lubitz SA, Mahida S, Magnani JW, Fontes JD, Sinner M, Van Gelder I, Ellinor P, Benjamin E (2012). Symptoms and functional status of patients with atrial fibrillation state of the art and future research opportunities. Circulation.

[6] Gallagher C, Hendriks JM, Giles L, Karnon J, Pham C, Elliott AD, Middeldorp ME, Mahajan R, Lau DH, Sanders P, Wong CX (2019). Increasing trends in hospitalisations due to atrial fibrillation in Australia from 1993 to 2013. Heart.

[7] Wong CX, Brooks AG, Leong DP, Roberts-Thomson KC, Sanders P (2012). The increasing burden of atrial fibrillation compared to heart failure and myocardial infarction: a 15-year study of all hospitalizations in Australia. Arch Intern Med.

[8] Tu SJ, Hanna-Rivero N, Elliott AD, Clarke N, Huang S, Pitman BM, Gallagher C, Linz D, Mahajan R, Lau DH, Sanders P, Wong CX (2021). Associations of anemia with stroke, bleeding, and mortality in atrial fibrillation: a systematic review and meta-analysis. J Cardiovasc Electrophysiol.

[9] Bonde AN, Blanche P, Staerk L, Gerds TA, Gundlund A, Gislason G, Torp-Pedersen C, Lip GYH, Hlatky MA, Olesen JB (2019). Oral anticoagulation among atrial fibrillation patients with anemia: an observational cohort study. Eur Heart J.

[10] Westenbrink BD, Alings M, Connolly SJ, Eikelboom J, Ezekowitz MD, Oldgren J, Yang S, Pongue J, Yusuf S, Wallentin L, van Gilst WH (2015). Anemia predicts thromboembolic events, bleeding complications and mortality in patients with atrial fibrillation: insights from the RE-LY trial. J Thromb Haemost.

[11] Westenbrink BD, Alings M, Granger CB, Alexander JH, Lopes RD, Hylek EM, Thomas L, Wojdyla DM, Hanna M, Keltai M, Steg PG, De Caterina R, Wallentin L, van Gilst WH (2017). Anemia is associated with bleeding and mortality, but not stroke, in patients with atrial fibrillation: insights from the Apixaban for Reduction in Stroke and Other Thromboembolic Events in Atrial Fibrillation (ARISTOTLE) trial. Am Heart J.

[12] Woo-Hyun L, Eue-Keun C, Kyung-Do H, So-Ryoung L, Myung-Jin C, Seil O (2020). Impact of hemoglobin levels and their dynamic changes on the risk of atrial fibrillation: a nationwide population-based study. Sci Rep.

[13] Xu DZ, Murakoshi N, Sairenchi T, Irie F, Igarashi M, Tomizawa T, Yamaguchi I, Iso H, Ota H, Aonuma K (2013). Reduced kidney function and anemia as risk factors for new onset AF. Eur Heart J.

[14] Kim M, Hong M, Kim JY, Kim IS, Yu HT, Kim TH, Uhm JS, Joung B, Lee MH, Pak HN (2020). Clinical relationship between anemia and atrial fibrillation recurrence after catheter ablation without genetic background. Int J Cardiol Heart Vasc.

[15] Von Haehling S, Jankowska E, Anker S (2011). Anemia in heart failure: an overview of current concepts. Future Cardiol.

[16] Wong CCY, Ng ACC, Kritharides L, Sindone AP (2016). Iron deficiency in heart failure: looking beyond anemia. Heart Lung Circ.

[17] Jankowska EA, Rozentryt P, Witkowska A, Nowak J, Hartmann O, Ponikowska B, Borodulin-Nadzieja L, Banasiak W, Polonski L, Filippatos G, McMurray JJV, Anker SD, Ponikowski P (2010). Iron deficiency: an ominous sign in patients with systolic chronic heart failure. Eur Heart J.

[18] Ponikowski P, Voors AA, Anker SD, Bueno H, Cleland JGF, Coats AJS, Falk V, González-Juanatey JR, Harjola V-P, Jankowska EA, Jessup M, Linde C, Nihoyannopoulos P, Parissis JT, Pieske B, Riley JP, Rosano GMC, Ruilope LM, Ruschitzka F, Rutten FH, van der Meer P, Filippatos G, McMurray JJV, Aboyans V, Achenbach S, Agewall S, Al-Attar N, Atherton JJ, Bauersachs J, John Camm A, Carerj S, Ceconi C, Coca A, Elliott P, Erol Ç, Ezekowitz J, Fernández-Golfín C, Fitzsimons D, Guazzi M, Guenoun M, Hasenfuss G, Hindricks G, Hoes AW, Iung B, Jaarsma T, Kirchhof P, Knuuti J, Kolh P, Konstantinides S, Lainscak M, Lancellotti P, Lip GYH, Maisano F, Mueller C, Petrie MC, Piepoli MF, Priori SG, Torbicki A, Tsutsui H, van Veldhuisen DJ, Windecker S, Yancy C, Zamorano JL, Badimon L, Barón-Esquivias G, Baumgartner H, Bax JJ, Dean V, Gaemperli O, Roffi M, Vaz Carneiro A, Sisakian HS, Isayev E, Kurlianskaya A, Mullens W, Tokmakova M, Agathangelou P, Melenovsky V, Wiggers H, Hassanein M, Uuetoa T, Lommi J, Kostovska ES, Juillière Y, Aladashvili A, Luchner A, Chrysohoou C, Nyolczas N, Thorgeirsson G, Marc Weinstein J, Di Lenarda A, Aidargaliyeva N, Bajraktari G, Beishenkulov M, Kamzola G, Abdel-Massih T, Čelutkienė J, Noppe S, Cassar A, Vataman E (2016). 2016 ESC Guidelines for the diagnosis and treatment of acute and chronic heart failure. Eur Heart J.

[19] Odutayo A, Wong C, Hsiao A, Hopewell S, Altman DG, Emdin C (2016). Atrial fibrillation and risks of cardiovascular disease, renal disease, and death: systematic review and meta-analysis. BMJ Br Med J.

[20] Lau DH, Kalman J, Sanders P (2014). Management of recent-onset sustained atrial fibrillation: pharmacologic and nonpharmacologic strategies. Clin Ther.

[21] Hohnloser SH (2011). Stroke prevention versus bleeding risk in atrial fibrillation: a clinical dilemma. J Am Coll Cardiol.

[22] Gleason KT, Nazarian S, Dennison Himmelfarb CR (2018). Atrial fibrillation symptoms and sex, race, and psychological distress: a literature review. J Cardiovasc Nurs.

[23] Sears SF, Serber ER, Alvarez LG, Schwartzman DS, Hoyt RH, Ujhelyi MR (2005). Understanding atrial symptom reports: objective versus subjective predictors. Pacing Clin Electrophysiol.

[24] Goette A, Bukowska A, Dobrev D, Pfeiffenberger J, Morawietz H, Strugala D, Wiswedel I, Rohl FW, Wolke C, Bergmann S, Bramlage P, Ravens U, Lendeckel U (2009). Acute atrial tachyarrhythmia induces angiotensin II type 1 receptor-mediated oxidative stress and microvascular flow abnormalities in the ventricles. Eur Heart J.

[25] Clark DM, Plumb VJ, Epstein AE, Kay GN (1997). Hemodynamic effects of an irregular sequence of ventricular cycle lengths during atrial fibrillation. J Am Coll Cardiol.

[26] Daoud EG, Weiss R, Bahu M, Knight BP, Bogun F, Goyal R, Harvey M, Strickberger SA, Man KC, Morady F (1996). Effect of an irregular ventricular rhythm on cardiac output. Am J Cardiol.

[27] Lau CP, Leung WH, Wong CK, Cheng CH (1990). Haemodynamics of induced atrial fibrillation: a comparative assessment with sinus rhythm, atrial and ventricular pacing. Eur Heart J.

[28] Skinner NS, Mitchell JH, Wallace AG, Sarnoff SJ (1964). Hemodynamic consequences of atrial fibrillation at constant ventricular rates. Am J Med.

[29] Metivier F, Marchais SJ, Guerin AP, Pannier B, London GM (2000). Pathophysiology of anemia: focus on the heart and blood vessels. Nephrol Dial Transplant.

[30] Singh SN, Tang XC, Singh BN, Dorian P, Reda DJ, Harris CL, Fletcher RD, Sharma SC, Atwood JE, Jacobson AK, Lewis HD, Lopez B, Raisch DW, Ezekowitz MD (2006). Quality of life and exercise performance in patients in sinus rhythm versus persistent atrial fibrillation. J Am Coll Cardiol.

[31] Swank AM, Horton J, Fleg JL, Fonarow GC, Keteyian S, Goldberg L, Wolfel G, Handberg EM, Bensimhon D, Illiou MC, Vest M, Ewald G, Blackburn G, Leifer E, Cooper L, Kraus WE (2012). Modest increase in peak VO_2_ is related to better clinical outcomes in chronic heart failure patients: results from heart failure and a controlled trial to investigate outcomes of exercise training. Circ Heart Fail.

[32] Vanhees L, Schepers D, Defoor J, Brusselle S, Tchursh N, Fagard R (2000). Exercise performance and training in cardiac patients with atrial fibrillation. J Cardiopulm Rehabil.

[33] Hegbom F, Stavem K, Sire S, Heldal M, Orning OM, Gjesdal K (2007). Effects of short-term exercise training on symptoms and quality of life in patients with chronic atrial fibrillation. Int J Cardiol.

[34] Katsumata Y, Tamura Y, Kimura T, Kohsaka S, Sadahiro T, Nishiyama T, Aizawa Y, Azuma K, Fukuda K, Takatsuki S (2019). A high BNP level predicts an improvement in exercise tolerance after a successful catheter ablation of persistent atrial fibrillation. J Cardiovasc Electrophysiol.

[35] Mohanty S, Santangeli P, Mohanty P, Biase LD, Holcomb S, Trivedi C, Bai R, Burkhardt D, Hongo R, Hao S, Beheiry S, Santoro F, Forleo G, Gallinghouse JG, Horton R, Sanchez JE, Bailey S, Hranitzky PM, Zagrodzky J, Natale A (2014). Catheter ablation of asymptomatic longstanding persistent atrial fibrillation: impact on quality of life, exercise performance, arrhythmia perception, and arrhythmia-free survival. J Cardiovasc Electrophysiol.

[36] Singh BN, Singh SN, Reda DJ, Tang XC, Lopez B, Harris CL, Fletcher RD, Sharma SC, Atwood JE, Jacobson AK, Lewis HD, Raisch DW, Ezekowitz MD (2005). Amiodarone versus sotalol for atrial fibrillation. N Engl J Med.

[37] Australian health survey: biomedical results for chronic diseases, 2011–12; 2013.

[38] McLean E, Cogswell M, Egli I, Wojdyla D, de Benoist B (2009). Worldwide prevalence of anemia, WHO vitamin and mineral nutrition information system, 1993–2005. Public Health Nutr.

[39] Goodnough LT, Schrier SL (2014). Evaluation and management of anemia in the elderly. Am J Hematol.

[40] Guralnik JM, Eisenstaedt RS, Ferrucci L, Klein HG, Woodman RC (2004). Prevalence of anemia in persons 65 years and older in the United States: evidence for a high rate of unexplained anemia. Blood.

[41] Pasricha SR, Flecknoe-Brown SC, Allen KJ, Gibson PR, McMahon LP, Olynyk JK, Roger SD, Savoia HF, Tampi R, Thomson AR, Wood EM, Robinson KL (2010). Diagnosis and management of iron deficiency anemia: a clinical update. Med J Aust.

[42] Adou P, Davidsson L, Cook J, Harrell R (2001). Prevalence of iron deficiency with and without concurrent anemia in population groups with high prevalences of malaria and other infections: a study in Cote d'Ivoire. Am J Clin Nutr.

[43] Callander EJ, Schofield DJ (2016). Is there a mismatch between who gets iron supplementation and who needs it? A cross-sectional study of iron supplements, iron deficiency anemia and socio-economic status in Australia. Br J Nutr.

[44] Ahmed F, Coyne T, Dobson A, McClintock C (2008). Iron status among Australian adults: findings of a population based study in Queensland, Australia. Asia Pac J Clin Nutr.

[45] Vanarsa K, Ye Y, Han J, Xie C, Mohan C, Wu T (2012). Inflammation associated anemia and ferritin as disease markers in SLE. Arthritis Res Ther.

[46] Tran TN, Eubanks SK, Schaffer KJ, Zhou CY, Linder MC (1997). Secretion of ferritin by rat hepatoma cells and its regulation by inflammatory cytokines and iron. Blood.

[47] Diagnosis and investigation of iron deficiency anemia. 2017.

[48] Rocha BML, Cunha GJL, Menezes Falcão LF (2018). The burden of iron deficiency in heart failure: therapeutic approach. J Am Coll Cardiol.

[49] Ponikowski P, Voors AA, Anker SD, Bueno H, Cleland JGF, Coats AJS, Falk V, González-Juanatey JR, Harjola V-P, Jankowska EA, Jessup M, Linde C, Nihoyannopoulos P, Parissis JT, Pieske B, Riley JP, Rosano GMC, Ruilope LM, Ruschitzka F, Rutten FH, van der Meer P, Group ESD. 2016 ESC Guidelines for the diagnosis and treatment of acute and chronic heart failure: The Task Force for the diagnosis and treatment of acute and chronic heart failure of the European Society of Cardiology (ESC) Developed with the special contribution of the Heart Failure Association (HFA) of the ESC. Eur Heart J. 2016;37:2129–2200.

[50] Von Haehling S, Ebner N, Evertz R, Ponikowski P, Anker SD (2019). Iron deficiency in heart failure. JACC Heart Fail.

[51] Beale AL, Warren JL, Roberts N, Meyer P, Townsend NP, Kaye D (2019). Iron deficiency in heart failure with preserved ejection fraction: a systematic review and meta-analysis. Open Heart.

[52] Pozzo J, Fournier P, Delmas C, Vervueren P-L, Roncalli J, Elbaz M, Galinier M, Lairez O (2017). Absolute iron deficiency without anemia in patients with chronic systolic heart failure is associated with poorer functional capacity. Arch Cardiovasc Dis.

[53] Van Veldhuisen DJ, Ponikowski P, van Der Meer P, Metra M, Böhm M, Doletsky A, Voors AA, Macdougall IC, Anker SD, Roubert B, Zakin L, Cohen-Solal A (2017). Effect of ferric carboxymaltose on exercise capacity in patients with chronic heart failure and iron deficiency. Circulation.

[54] Anker SD, Kirwan BA, van Veldhuisen DJ, Filippatos G, Comin-Colet J, Ruschitzka F, Lüscher TF, Arutyunov GP, Motro M, Mori C, Roubert B, Pocock SJ, Ponikowski P (2018). Effects of ferric carboxymaltose on hospitalisations and mortality rates in iron-deficient heart failure patients: an individual patient data meta-analysis. Eur J Heart Fail.

[55] Ponikowski P, Kirwan BA, Anker SD, McDonagh T, Dorobantu M, Drozdz J, Fabien V, Filippatos G, Gohring UM, Keren A, Khintibidze I, Kragten H, Martinez FA, Metra M, Milicic D, Nicolau JC, Ohlsson M, Parkhomenko A, Pascual-Figal DA, Ruschitzka F, Sim D, Skouri H, van der Meer P, Lewis BS, Comin-Colet J, von Haehling S, Cohen-Solal A, Danchin N, Doehner W, Dargie HJ, Motro M, Butler J, Friede T, Jensen KH, Pocock S, Jankowska EA, investigators A-A. Ferric carboxymaltose for iron deficiency at discharge after acute heart failure: a multicentre, double-blind, randomised, controlled trial. Lancet. 2020;396:1895–1904. 10.1016/S0140-6736(20)32339-4 33197395

[56] Weiss G, Goodnough LT (2005). Anemia of chronic disease. N Engl J Med.

[57] Okonko DO, Mandal AKJ, Missouris CG, Poole-Wilson PA (2011). Disordered iron homeostasis in chronic heart failure. J Am Coll Cardiol.

[58] Nemeth E, Rivera S, Gabayan V, Keller C, Taudorf S, Pedersen BK, Ganz T (2004). IL-6 mediates hypoferremia of inflammation by inducing the synthesis of the iron regulatory hormone hepcidin. J Clin Investig.

[59] Jelkmann W (1998). Proinflammatory cytokines lowering erythropoietin production. J Interferon Cytokine Res.

[60] Lofruthe N, Gallitz I, Traeger L, Baumer N, Schulze I, Kuhlmann T, Muller-Tidow C, Steinbicker AU (2016). Intravenous iron carboxymaltose as a potential therapeutic in anemia of inflammation. PLoS ONE.

[61] McDonagh T, Macdougall IC (2015). Iron therapy for the treatment of iron deficiency in chronic heart failure: intravenous or oral?. Eur J Heart Fail.

[62] Maisel WH, Stevenson LW (2003). Atrial fibrillation in heart failure: epidemiology, pathophysiology, and rationale for therapy. Am J Cardiol.

[63] Hu Y-F, Chen Y-J, Lin Y-J, Chen S-A (2015). Inflammation and the pathogenesis of atrial fibrillation. Nat Rev Cardiol.

[64] Schwartz AJ, Converso-Baran K, Michele DE, Shah YM (2019). A genetic mouse model of severe iron deficiency anemia reveals tissue-specific transcriptional stress responses and cardiac remodeling. J Biol Chem.

[65] Puurunen M, Kiviniemi T, Nammas W, Schlitt A, Rubboli A, Nyman K, Karjalainen P, Kirchhof P, Lip GYH, Airaksinen JKE (2014). Impact of anemia on clinical outcome in patients with atrial fibrillation undergoing percutaneous coronary intervention: insights from the AFCAS registry. BMJ Open.

[66] Westenbrink BD, van Gilst WH, Lopes RD, Thomas L, Wojdyla DM (2017). Anemia is associated with bleeding and mortality, but not stroke, in patients with atrial fibrillation: Insights from the Apixaban for Reduction in Stroke and Other Thromboembolic Events in Atrial Fibrillation (ARISTOTLE) trial. Am Heart J.

[67] Krittayaphong R, Pumprueg S, Thongsri T, Wiwatworapan W, Choochunklin T, Kaewkumdee P, Yindeengam A, Investigators C-A (2021). Impact of anemia on clinical outcomes of patients with atrial fibrillation: the COOL-AF registry. Clin Cardiol.

[68] Hashimoto K, Kimura T, Ikemura N, Katsumata Y, Fujisawa T, Miyama H, Yamashita T, Nakamura I, Mano Y, Oki T, Fukuda K, Kohsaka S, Takatsuki S (2021). Burden of mild (<13 g/dl) anemia in patients with atrial fibrillation (a report from a multicenter registry with patient-reported outcomes). Am J Cardiol.

[69] An Y, Ogawa H, Esato M, Ishii M, Iguchi M, Masunaga N, Fujino A, Ide Y, Hamatani Y, Doi K, Ikeda S, Ishigami K, Tsuji H, Wada H, Hasegawa K, Abe M, Lip GYH, Akao M, Fushimi AFRI. Cardiovascular events and mortality in patients with atrial fibrillation and anemia (from the Fushimi AF registry). Am J Cardiol. 2020;134:74–82. 10.1016/j.amjcard.2020.08.009 32900468

[70] Bassand J-P, Accetta G, Camm AJ, Cools F, Fitzmaurice DA, Fox KAA, Goldhaber SZ, Goto S, Haas S, Hacke W, Kayani G, Mantovani LG, Misselwitz F, Ten Cate H, Turpie AGG, Verheugt FWA, Kakkar AK, Investigators G-A (2016). Two-year outcomes of patients with newly diagnosed atrial fibrillation: results from GARFIELD-AF. Eur Heart J.

[71] An Y, Ogawa H, Yamashita Y, Ishii M, Iguchi M, Masunaga N, Esato M, Tsuji H, Wada H, Hasegawa K, Abe M, Lip GYH, Akao M (2019). Causes of death in Japanese patients with atrial fibrillation: the Fushimi Atrial Fibrillation Registry. Eur Heart J Qual Care Clin Outcomes.

[72] Anker SD, Voors A, Okonko D, Clark AL, James MK, von Haehling S, Kjekshus J, Ponikowski P, Dickstein K, for the OI. Prevalence, incidence, and prognostic value of anemia in patients after an acute myocardial infarction: data from the OPTIMAAL trial. Eur Heart J. 2009;30:1331–1339. 10.1093/eurheartj/ehp116 19383732

[73] Keskin M, Ural D, Altay S, Argan O, Börklü EB, Kozan Ö (2018). Iron deficiency and hematinic deficiencies in atrial fibrillation: a new insight into comorbidities. Turk Kardiyoloji Dernegi arsivi: Turk Kardiyoloji Derneginin yayin organidir.

[74] Imai R, Higuchi T, Morimoto M, Koyamada R, Okada S (2018). Iron deficiency anemia due to the long-term use of a proton pump inhibitor. Internal Med (Tokyo, Japan).

[75] Lopez A, Cacoub P, Macdougall IC, Peyrin-Biroulet L (2016). Iron deficiency anemia. Lancet.

[76] Minhas AMK, Sagheer S, Shekhar R, Sheikh AB, Nazir S, Ullah W, Khan MZ, Shahid I, Dani SS, Michos ED, Fudim M. Trends and inpatient outcomes of primary atrial fibrillation hospitalizations with underlying iron deficiency anemia: an analysis of the national inpatient sample database from 2004–2018. Curr Probl Cardiol. 2021;101001. 10.1016/j.cpcardiol.2021.101001 34571106

[77] Shireman TI, Mahnken JD, Howard PA, Kresowik TF, Hou Q, Ellerbeck EF (2006). Development of a contemporary bleeding risk model for elderly warfarin recipients. Chest.

[78] Sharma S, Gage BF, Deych E, Rich MW (2009). Anemia: an independent predictor of death and hospitalizations among elderly patients with atrial fibrillation. Am Heart J.

[79] Park SJ, Lee SC, Jang SY, Chang SA, Choi JO, Park SW, Oh JK (2011). E/e' ratio is a strong prognostic predictor of mortality in patients with non-valvular atrial fibrillation with preserved left ventricular systolic function. Circ J.

[80] Suzuki S, Sagara K, Otsuka T, Matsuno S, Funada R, Uejima T, Oikawa Y, Yajima J, Koike A, Nagashima K, Kirigaya H, Sawada H, Aizawa T, Yamashita T (2012). A new scoring system for evaluating the risk of heart failure events in japanese patients with atrial fibrillation. Am J Cardiol.

[81] Lip GY, Banerjee A, Lagrenade I, Lane DA, Taillandier S, Fauchier L (2012). Assessing the risk of bleeding in patients with atrial fibrillation: the Loire Valley Atrial Fibrillation project. Circ Arrhythm Electrophysiol.

[82] Friberg L, Rosenqvist M, Lip GY (2012). Evaluation of risk stratification schemes for ischemic stroke and bleeding in 182 678 patients with atrial fibrillation: the Swedish Atrial Fibrillation cohort study. Eur Heart J.

[83] Manzano-Fernandez S, Cambronero F, Caro-Martinez C, Hurtado-Martinez JA, Marin F, Pastor-Perez FJ, Mateo-Martinez A, Sanchez-Martinez M, Pinar-Bermudez E, Valdes M (2012). Mild kidney disease as a risk factor for major bleeding in patients with atrial fibrillation undergoing percutaneous coronary stenting. Thromb Haemost.

[84] Takabayashi K, Takagi D, Hamatani Y, Unoki T, Esato M, Chun Y, Wada H, Hasegawa K, Abe M, Akao M (2014). Incidence of hospitalization for heart failure in atrial fibrillation patients with anemia: one-year follow-up of the Fushimi AF Registry. Eur Heart J.

[85] Goodman SG, Wojdyla DM, Piccini JP, White HD, Paolini JF, Nessel CC, Berkowitz SD, Mahaffey KW, Patel MR, Sherwood MW, Becker RC, Halperin JL, Hacke W, Singer DE, Hankey GJ, Breithardt G, Fox KA, Califf RM, Investigators RA (2014). Factors associated with major bleeding events: insights from the ROCKET AF trial (rivaroxaban once-daily oral direct factor Xa inhibition compared with vitamin K antagonism for prevention of stroke and embolism trial in atrial fibrillation). J Am Coll Cardiol.

[86] Sherwood MW, Nessel CC, Hellkamp AS, Mahaffey KW, Piccini JP, Suh EY, Becker RC, Singer DE, Halperin JL, Hankey GJ, Berkowitz SD, Fox KAA, Patel MR (2015). Gastrointestinal bleeding in patients with atrial fibrillation treated with rivaroxaban or warfarin: ROCKET AF trial. J Am Coll Cardiol.

[87] Lee WH, Hsu PC, Chu CY, Lee HH, Lee MK, Lee CS, Yen HW, Lin TH, Voon WC, Lai WT, Sheu SH, Su HM (2015). Anemia as an independent predictor of adverse cardiac outcomes in patients with atrial fibrillation. Int J Med Sci.

[88] O'Brien EC, Simon DN, Thomas LE, Hylek EM, Gersh BJ, Ansell JE, Kowey PR, Mahaffey KW, Chang P, Fonarow GC, Pencina MJ, Piccini JP, Peterson ED (2015). The ORBIT bleeding score: a simple bedside score to assess bleeding risk in atrial fibrillation. Eur Heart J.

[89] Dodson JA, Petrone A, Gagnon DR, Tinetti ME, Krumholz HM, Gaziano JM (2016). Incidence and determinants of traumatic intracranial bleeding among older veterans receiving warfarin for atrial fibrillation. JAMA Cardiol.

[90] Hori M, Matsumoto M, Tanahashi N, Momomura SI, Uchiyama S, Goto S, Izumi T, Koretsune Y, Kajikawa M, Kato M, Cavaliere M, Iekushi K, Yamanaka S, Investigators JRAS (2016). Predictive factors for bleeding during treatment with rivaroxaban and warfarin in Japanese patients with atrial fibrillation - Subgroup analysis of J-ROCKET AF. J Cardiol.

[91] Kobayashi N, Yamawaki M, Nakano M, Hirano K, Araki M, Takimura H, Sakamoto Y, Mori S, Tsutsumi M, Ito Y (2016). A new scoring system (DAIGA) for predicting bleeding complications in atrial fibrillation patients after drug-eluting stent implantation with triple antithrombotic therapy. Int J Cardiol.

[92] Li G, Thabane L, Delate T, Witt DM, Levine MA, Cheng J, Holbrook A (2016). Can we predict individual combined benefit and harm of therapy? warfarin therapy for atrial fibrillation as a test case. PLoS ONE.

[93] Aisenberg J, Chatterjee-Murphy P, Friedman Flack K, Weitz JI, Ruff CT, Nordio F, Mercuri MF, Choi Y, Antman EM, Braunwald E, Giugliano RP (2018). Gastrointestinal bleeding with edoxaban versus warfarin: results from the ENGAGE AF-TIMI 48 trial (effective anticoagulation with factor Xa next generation in atrial fibrillation-thrombolysis in myocardial infarction). Circ Cardiovasc Qual Outcomes.

[94] Perera KS, Pearce LA, Sharma M, Benavente O, Connolly SJ, Hart RG, Committee AAS, Investigators. Predictors of mortality in patients with atrial fibrillation (from the atrial fibrillation clopidogrel trial with irbesartan for prevention of vascular events [ACTIVE A]). Am J Cardiol. 2018;121:584–589. 10.1016/j.amjcard.2017.11.028 29291887

[95] An Y, Iguchi M, Ishii M, Masunaga N, Aono Y, Ikeda S, Ishigami K, Doi K, Esato M, Tsuji H, Wada H, Hasegawa K, Ogawa H, Abe M, Akao M (2019). Prognostic impact of anemia on stroke/systemic embolism, bleeding, and mortality in patients with atrial fibrillation: the fushimi af registry. Circulation.

[96] Tiili P, Putaala J, Mehtala J, Khanfir H, Niiranen J, Korhonen P, Raatikainen P, Lehto M (2019). Poor quality of warfarin treatment increases the risk of all types of intracranial hemorrhage in atrial fibrillation. Circ J.

[97] Kuronuma K, Okumura Y, Yokoyama K, Matsumoto N, Tachibana E, Oiwa K, Matsumoto M, Kojima T, Hanada S, Nomoto K, Arima K, Takahashi F, Kotani T, Ikeya Y, Fukushima S, Itou S, Kondo K, Chiku M, Ohno Y, Onikura M, Hirayama A, Investigators SAR (2019). Different determinants of vascular and nonvascular deaths in patients with atrial fibrillation: a SAKURA AF Registry substudy. J Cardiol.

[98] Fu S, Jiao J, Guo Y, Zhu B, Luo L (2019). N-terminal pro-brain natriuretic peptide levels had an independent and added ability in the evaluation of all-cause mortality in older Chinese patients with atrial fibrillation. BMC Geriatr.

[99] Kodani E, Inoue H, Atarashi H, Okumura K, Yamashita T, Origasa H (2020). Impact of hemoglobin concentration and platelet count on outcomes of patients with non-valvular atrial fibrillation: a subanalysis of the J-RHYTHM Registry. Int J Cardiol.

[100] Zhang Z, Jiang C, He L, Bai Y, Wu J, Hu R, Lv Q, Ning M, Feng L, Tang R, Sang C, Long D, Dong J, Du X, Lip GYH, Ma C (2022). Associations of anemia with death and major bleeding in patients with atrial fibrillation: a report from the Chinese Atrial Fibrillation Registry Study. Clin Cardiol.

[101] Al-Hussainy N, Kragholm KH, Lundbye-Christensen S, Torp-Pedersen C, Pareek M, Therkelsen SK, Lip GYH, Riahi S (2021). Safety and efficacy of direct oral anticoagulants (DOAC) in patients with anemia and atrial fibrillation: an observational nation-wide Danish cohort study. Eur Heart J Qual Care Clin Outcomes.

